# Uptake of video telehealth in general practice: an Australian whole-of-population analysis

**DOI:** 10.3399/BJGPO.2025.0011

**Published:** 2025-12-19

**Authors:** Danielle C Butler, Hsei Di Law, Christine Phillips, Kirsty A Douglas, Sally Hall Dykgraaf, Jason Agostino, Emily Banks, Rachel Freeman-Robinson, Jane Desborough, Alana Dougherty, Grace Joshy, Nina Lazarevic, Jennifer Welsh, Muhammad-Shahdaat Bin-Sayeed, Dan Chateau, Kay Soga, Anne Parkinson, Sue Trevenar, Rosemary J Korda

**Affiliations:** 1 National Centre for Epidemiology and Population Health, Australian National University, Canberra, Australia; 2 School of Medicine and Psychology, Australian National University, Canberra, Australia; 3 Health and Aboriginal and Torres Strait Islander Statistics Branch, Australian Bureau of Statistics, Brisbane, Australia; 4 Australian Institute for Health and Welfare, Canberra, Australia

**Keywords:** general practice, health policy, linked data, telehealth, telemedicine

## Abstract

**Background:**

Video use remains low in primary care telehealth consultations. Little is known about patterns of use or policy levers to promote video.

**Aim:**

To investigate use of video telehealth in Australian general practice under permanent telehealth arrangements post-COVID-19 lockdowns, and during a policy change removing reimbursement for long telephone consultations.

**Design & setting:**

Whole-of-population analysis of 2022 national healthcare claims linked to 2021 census data.

**Method:**

We quantified the following: proportions of telehealth consultations by video, and of patients and GPs who used video for telehealth consultations; associations between video use and patient characteristics using Poisson regression; and video use in relation to policy changes using interrupted time-series analysis.

**Results:**

Of 38 million GP telehealth consultations in 2022, 5.1% were by video; 8.6% of patients and 62% of GPs who used telehealth had used video. Patients most likely to use video lived remotely, were frequent GP users, or had multiple health conditions, mental health conditions or dementia. Socioeconomic disadvantage was modestly associated with lower use of video. Over 2022, use of video for telehealth decreased for consultations (from 6.5% of consultations in January to 4.1% in December), patients (from 6.7% to 4.4%), and GPs (from 40.0% to 26.0%). Time-series analyses showed downward trends before removal of reimbursement for long telephone consultations, small step increases immediately following, and shallower negative trends thereafter.

**Conclusion:**

Use of video telehealth consultations in general practice in Australia is low and declining, more so for disadvantaged groups. Differential financial reimbursement of video and telephone consultations has not substantively increased video use in clinical practice.

## How this fits in

Australian general practice has seen widescale adoption of telehealth, but ~5% of consultations are delivered by video, despite policy preference for this modality. Our study showed only around one in 10 patients, and nearly two in three GPs, using telehealth use video in 2022, with frequent GP users, those with mental health problems or dementia, and/or living with socioeconomic advantage being the most likely to use video. Video use declined over 2022, even after a policy change to remove reimbursement for longer telephone (but not video) consultations. Telephone may offer some advantages over video in Australian general practice and policies limiting telephone access are unlikely to substantively shift clinical practice to greater use of video.

## Introduction

The large-scale adoption of telehealth following the start of the COVID-19 pandemic was a major shift in primary care delivery globally.^1^ In Australia in 2020–2021, 23% of patient consultations with GPs, Australia’s main primary care providers, were conducted by remote synchronous telehealth, compared with <1% previously.^2^ More than 95% of these telehealth consultations were by telephone (that is, audio only) rather than video.

The low use of video telehealth in general practice, was unanticipated by policymakers. When temporary reimbursement for GP telehealth consultations was first introduced at the beginning of the pandemic under Medicare, Australia’s universal health insurance scheme, the intention was to subsidise video consultations as a substitute for face-to-face consultations, with telephone services to be used if video was not available.^3^ It was assumed that video consultations might carry lower clinical risk and provide higher quality of care than phone consultations,^3,4^ a policy position not unique to Australia.^5–7^

In January 2022, GP telehealth items were made permanent and in July of that year reimbursements for long (>20 minutes) telephone, but not video, consultations were removed, except in remote areas (Supplementary Box 1). Stakeholders raised concerns over possible effects of curtailing telephone consultations on access and equity of access to care.^3^ Moreover removing reimbursement for telephone consultations without considering how telehealth use varies across patient groups could exacerbate barriers to care already experienced by medically underserved and disadvantaged populations. In the US, for example, lower use of video versus telephone consultations in primary care among disadvantaged and minority populations has been documented.^7–10^ We lack Australian evidence on this, and more generally on changes in telehealth use by patients and providers when reimbursement for longer telephone consultations are restricted.

This study aimed to quantify use of video telehealth in Australian general practice under permanent telehealth arrangements and post-COVID-19 lockdowns, including by patient characteristics and in relation to a policy change to remove reimbursement for longer telephone consultations.

## Method

### Data sources

We used data compiled from the Person Level Integrated Data Asset (PLIDA) administered by the Australian Bureau of Statistics (ABS).^11^ This included national claims data from the Medicare Benefits Schedule (MBS) dataset for 2022, linked deterministically by the ABS to individual-level 2021 census data, using name, full date of birth, address, and sex. MBS data from 1 January 2020–31 December 2021 were used to define new users in 2022 and in supplementary analysis of historical trends.

The MBS dataset records all claims for medical services that are reimbursed under Medicare, which covers all Australian citizens and permanent residents. Variables include the date of service, de-identified patient and provider numbers distinguishing unique patients and doctors, and information on modality (in-person, telephone or video, see Table 1, Supplementary Box 1).

**Table 1.** Distribution of services by type of GP consultation, 2022

	In person		Audio		Video		Total
	*n*	%	*n*	%	*n*	%	*n*
**Total^a^**	125 943 463	76.8%	36 057 338	22.0%	1 948 880	1.2%	163 949 681
							
**Standard time-based services^b^**							
**A**	3 888 633	3.1%	4 417 390	12.3%	61 521	3.0%	8 367 544
**B**	75 433 066	59.9%	30 485 237	84.5%	962 141	49.4%	106 880 444
**C**	18 095 961	14.4%	690 007	1.9%	304 684	15.6%	19 090 652
**D**	1 589 182	1.3%	NA		59 369	3.2%	1 648 551
**Other^c^**	26 936 621	21.4%	464 704	1.3%	561 165	28.8%	27 962 490

^a^All items are those claimed by GPs or other medical practitioners (not including specialist or consultant physician) providing primary care services. ^b^Standard time-based consultations: A: attendance for an obvious problem or<6 minutes; B:<20 minutes; C:≥20 minutes; D:≥40 minutes. Cut-offs for categories B, C, and D are extended by 5 minutes for other medical practitioner in-person and video services. ^c^Other includes: extended primary care (including health assessments, chronic disease management, mental health services), sexual and reproductive health, pregnancy counselling, and nicotine and smoking cessation services. Other ‘in-person’ consultations include after-hours and residential aged care facility attendances.

Sociodemographic characteristics were from the 2021 census, which included usual residents of Australia on the night of 10 August 2021 with an estimated 95.8% person-response rate.^12^

### Study population

For each month of 2022, we included all MBS GP claims for services provided in that month, and the patients and GPs associated with those claims. Patients were defined as those who had at least one MBS GP service claim in that month. Active GPs were defined as providers for whom at least 50% of their total claims in that month were for GP services and who provided ≥20 GP services in that month.

**Figure 1.**
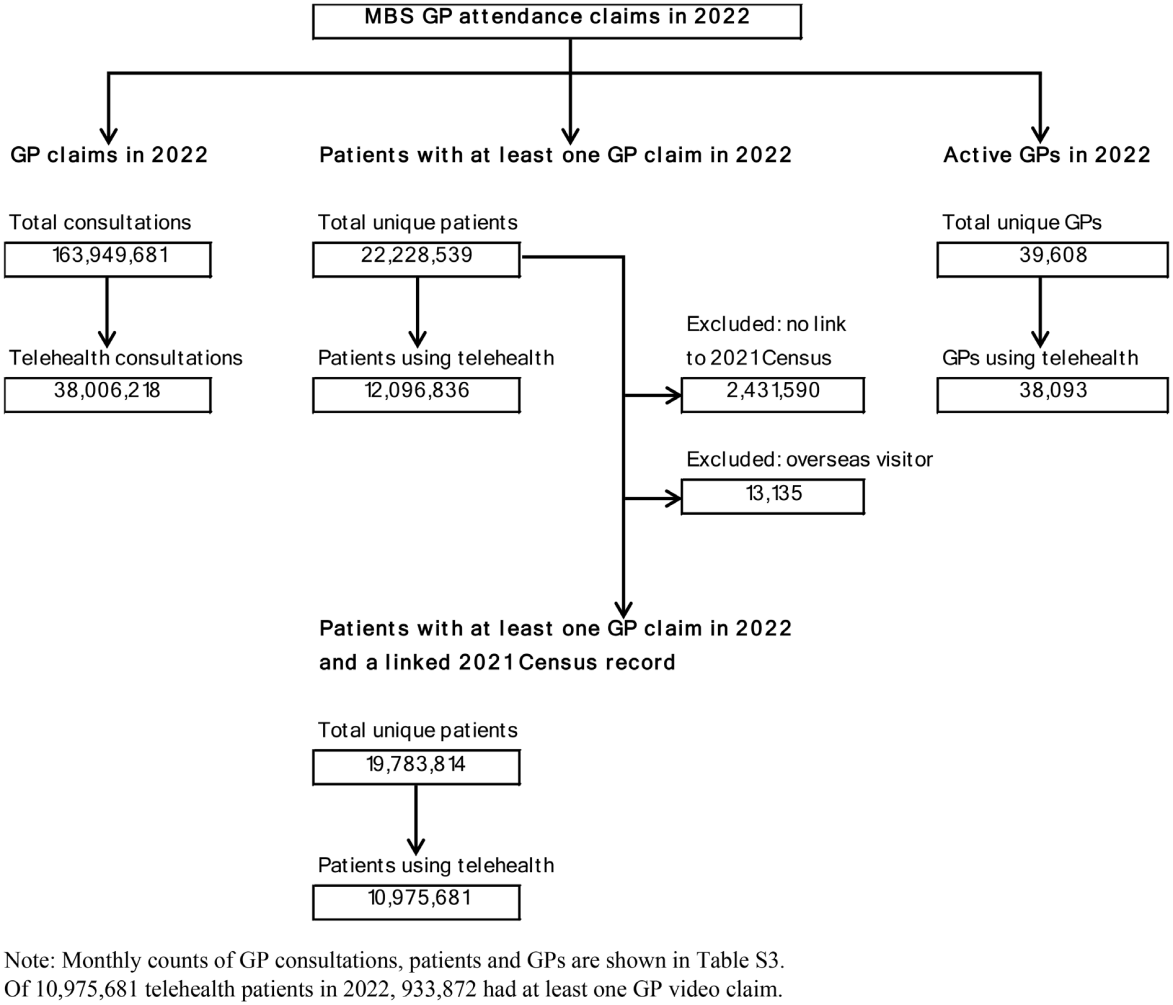
Flow diagram of study population. Note: monthly counts of GP consultations, patients, and GPs are shown in Table S3. Of 10 975 681 telehealth patients in 2022, 933 872 had at least one GP video claim

In analyses of patient characteristics associated with video use, we included only patients with at least one MBS GP service claim in 2022 and had a linked 2021 census record (Figure 1).

### Outcomes and variables

For each month and all of 2022 combined, we calculated the number of GP consultations, patients and active GPs by modality (in-person, telephone, or video). Study outcomes were the proportion of (a) GP telehealth consultations by video; (b) patients using GP telehealth services who used video at least once; and (c) GPs using telehealth who used video at least once. We also calculated video use as a proportion of all GP consultations and for patients and GPs, and the proportion of telehealth users (patients and GPs) in each month who used video for the first time (since January 2020). We also described overall telehealth use as a proportion of all GP consultations and for patients and GPs in 2022 to provide additional context.

For the analysis of patient characteristics associated with video use in 2022, characteristics included: age, sex, education, equivalised household income, area-level socioeconomic status, remoteness areas, marital status, English language proficiency, and self-reported long-term health conditions (Supplementary Table 2). We also grouped patients according to frequency of GP consultations, based on their number of MBS GP claims in 2022. In supplementary analyses, we also report on patient characteristics associated with overall telehealth use.

### Analysis

We used counts and proportions to describe outcomes. We used modified Poisson regression models to estimate relative risks (RR) and risk differences (RD) for video use, adjusting for age, sex and remoteness of residence. In supplementary analyses, we adjusted for (a) age and sex only; and (b) age, sex, remoteness, and frequency of GP consultations.

To examine outcomes over time, and specifically in relation to the policy change in July 2022 (when reimbursements for long telephone consultations were removed, except in remote areas), we conducted interrupted time-series analyses using segmented linear regression models with Newey–West standard errors adjusted for first-order autocorrelation. We estimated the rate of change (that is, slope) per month before the policy change (January 2022–June 2022), the change in slope following the policy change (July 2022–December 2022), as well as the instantaneous step change (June 2022–July 2022) when the policy change was introduced. We also performed separate analyses according to patient characteristics for those with a linked census record. While we focused our above analyses on the contemporary use of video in 2022 under relatively stabilised policy settings post-COVID-19 lockdowns, we also described changes over time in uptake before 2022 (April 2020–December 2021) in supplementary analysis.

Analyses were completed in DataLab, a secure remote access facility managed by the ABS, using SAS (version 9.4).

## Results

### Study population characteristics

In 2022, there were around 164 million GP consultations claimed (83% for standard time-based services, Table 1), just over 22 million unique patients who had at least one GP consultation and just under 40 000 active GPs (Figure 1). Per month, on average there were around 14 million consultations, 8 million patients, and 34 000 active GPs (Table 2, Supplementary Table 3a-b). A census record could be linked to MBS data for 89% of the sample (Figure 1, Supplementary Table 2).

**Table 2.** Monthly counts and proportions of patient consulting GPs, by modality, 2022

Month 2022	Patients consulting GPs, *n*	Using telehealth, *n*	Patients using telehealth, %	Using video telehealth, *n*	First-time video users, *n*	Patients using telehealth who used video, %
Jan	7 203 314	2 680 029	37.2	180 860	109 391	6.7
Feb	7 639 455	2 466 556	32.3	152 607	83 609	6.2
Mar	8 376 429	2 730 786	32.6	159 209	85 963	5.8
Apr	7 872 283	2 267 770	28.8	119 522	60 088	5.3
May	9 544 308	2 646 479	27.7	134 418	66 643	5.1
Jun	8 926 402	2 551 546	28.6	122 404	58 379	4.8
Jul	8 137 804	2 536 460	31.2	128 088	60 814	5.0
Aug	8 663 396	2 579 596	29.8	124 688	56 377	4.8
Sep	8 048 758	2 197 358	27.3	102 087	43 274	4.6
Oct	8 078 625	2 083 172	25.8	97 171	40 684	4.7
Nov	8 364 562	2 234 719	26.7	103 858	43 162	4.6
Dec	7 281 997	2 040 835	28.0	90 207	37 057	4.4
Jan–Dec	22 228 539	12 096 836	54.4	1 035 637	745 441	8.6

Monthly counts and proportion of GP consultations and GPs providing consultations, by modality, in 2022 are provided in Table S3

### Use of video telehealth in 2022

*Consultations*: Overall, 23% of GP consultations in 2022 were by telehealth (38 million), and 5.1% of these were via video (Supplementary Table 3a). The majority of video consultations were Level B (6 to <20 minutes, 49%) equivalent services (Table 1).

*Patients*: In 2022, 54% of patients using GP services used telehealth, and 8.6% of these patients used video at least once (Table 2). On average, 1.6% of patients consulting a GP in any month used video (5.2% of all patients using telehealth). Around half of the patients using video in any month were new users (range: 60% in January, 41% in December).

*GPs*: In 2022, 96% of active GPs used telehealth, and 62% of these GPs used video at least once (Supplementary Table 3b). On average, 33% of GPs who used telehealth in any month used video (median video consultations per GP = 4). Around 2% were first-time users (range: 4.5% in February, 1.1% in October).

### Variation in use of video telehealth by patient characteristics

Among the 19 783 814 patients with linked census data, 56% used telehealth during 2022; of these, 8.5% used video. The crude proportion using video was higher among people living in very remote versus other areas (15.1%) but otherwise varied modestly across sociodemographic subgroups, and was higher among frequent users of GP services (14.5% of people with ≥20 GP consultations) and among people with multiple long-term health conditions versus those without (Supplementary Table 2).

**Figure 2.**
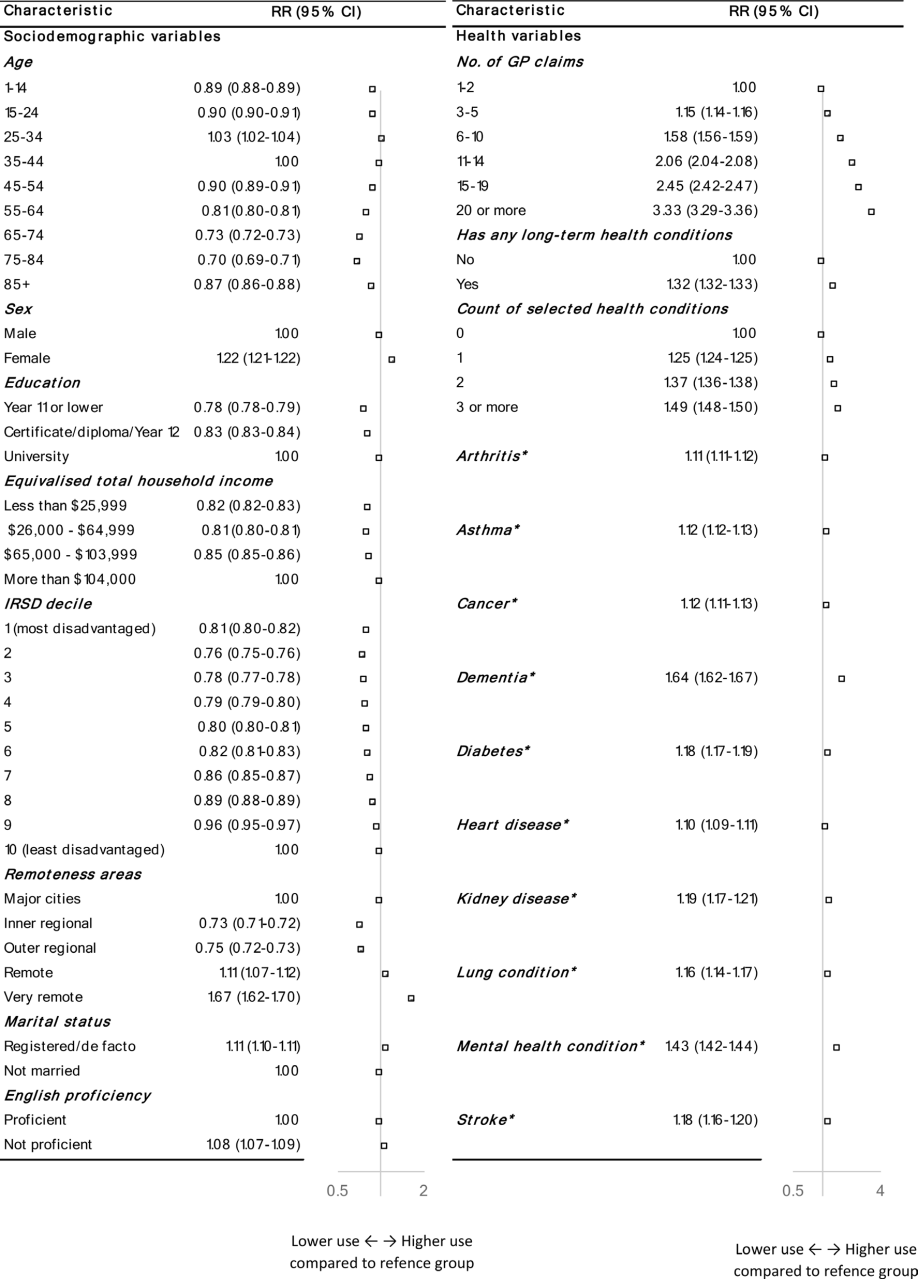
Associations between patient characteristics and any use of video telehealth among patients using GP telehealth services in 2022: relative risks (RR) adjusted for age, sex, and remoteness ^a^versus those who did not report the condition. 1. Age as reported on the 2021 census was incremented by 1 year to obtain age in 2022. 2. Forest plots show point estimates of adjusted RR (filled squares), associated 95% confidence interval (CI) interval (horizontal lines) and solid vertical line at RR equals one (reference group); values <1 indicate lower probability of using video than the reference group (values >1 indicate a higher probability). Adjusted RRs are on a log scale. 3. Raw numbers and risk differences (RDs) are presented in Table S2. IRSD = Index of Relative Socioeconomic Disadvantage

Compared with living in a major city, the probability of telehealth users using video was lower in inner and outer-regional areas and higher in remote or very remote areas, adjusting for age and sex. Adjusting for age, sex, and remoteness, lower video uptake was associated with male sex, older age, lower education, lower income, living in a more disadvantaged area, not being married (registered or de facto) and being proficient in English language, all with modest effect sizes (Figure 2, Supplementary Table 2). The probability of using video increased substantially with increasing frequency of GP consultations (for example, RR 3.33, 95% confidence interval [CI] = 3.29 to 3.36 for ≥20 versus 1–2 consultations) and with increasing number of health conditions (RR 1.49, 95% CI = 1.48 to 1.50 for ≥3 versus no conditions), and was relatively high for patients with dementia (RR 1.64, 95% CI = 1.62 to 1.67) and mental health conditions (RR 1.43, 95% CI = 1.42 to 1.44).

Results were broadly similar when adjusted for age and sex only, or for age, sex, remoteness, and frequency of GP consultations (Supplementary Table 4).

Similar patterns were seen for associations between patient characteristics and overall use of GP telehealth (video or telephone), with two exceptions. Older people were more likely than younger people to use telehealth, and people living outside major cities were less likely to use telehealth than those living in major cities (Supplementary Table 5).

### Changes over time in use of video telehealth

During 2022, there was a steady decline in proportion of patients using GP telehealth by video, from 6.7% in January to 4.4% in December. While the proportion of patients using GP services by telehealth also declined over 2022, use of video declined proportionally more than telephone (Table 2). The same pattern was seen for GP telehealth consultations by video (6.5% down to 4.4%), and for GPs using video (40% down to 26%) (Supplementary Table 3a&b). For changes in uptake before 2022 see Supplementary Table 7.

**Figure 3.**
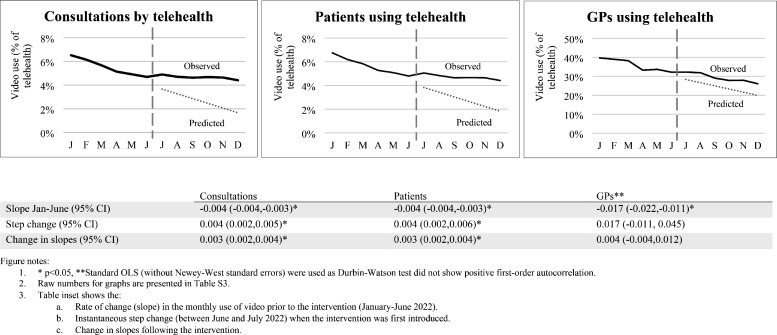
Time-series analysis: monthly trends in use of video (as a proportion of telehealth) before and after July 2022 policy change that removed reimbursements for standard long consultation items by telephone * *P*<0.05, **Standard ordinary least squares (OLS) (without Newey–West standard errors) were used as Durbin–Watson test did not show positive first-order autocorrelation. 1. Raw numbers for graphs are presented in Table S3. 2. Table inset shows the: a. Rate of change (slope) in the monthly use of video before the intervention (January–June 2022). b. Instantaneous step change (between June and July 2022) when the intervention was first introduced. c. Change in slopes following the intervention

Interrupted time-series analyses for consultations, patients, and GPs (Figure 3) showed: significant downward trends in use of video before the policy change (January 2022–June 2022, *P*<0.05); small step increases in video consultations and patient use of video immediately following the policy change (June–July); and shallower trends after the policy change. Trends were similar across population groups with few exceptions (Supplementary Table 6).

In terms of changes in overall use of telehealth, the proportion of GP consultations by telehealth also declined over 2022. This decline was greater for long (>20 minute) consultations, falling from 8.2% in January–June (before the policy change) to 1.7% in July–December (79% reduction), than for short (≤20 minute) consultations, which fell from 32% to 30% (7% reduction). At the same time, long GP consultations (all modalities) constituted 15% of all GP consultations in both the 6 months before and after the policy change (Supplementary Table 3c).

## Discussion

### Summary

In the third year (2022) of the telehealth roll-out in Australia and with telehealth reimbursement made permanent, nearly all GPs used telehealth, and almost one in four consultations were by telehealth. However, only 5% of telehealth consultations were by video and more than 90% of telehealth patients and more than one-third of GPs did not use video at all. Even among GPs using video, the majority used it infrequently (<5 times per month).

While the policy change to limit use of telephone consultations to shorter consultations in mid-2022 was initially associated with small increases in video consultations and in patients using video, this was not sustained. The small changes partly reflect that the policy only affected long consultations. That the proportion of long consultations delivered by telehealth declined dramatically after the policy change (from >8% to <2%), while the overall proportion of GP consultations that were long remained stable, indicates a shift to in-person rather than video consultations. Patient use of video continued to fall after the policy change, albeit at a slower rate, a pattern seen across most population subgroups. The decline in the proportion of GPs using video also continued throughout 2022 and very few GPs who had not used video before took it up after the policy change. Taken together, this suggests that current policy settings are unlikely to substantively shift clinical practice to greater use of video.

### Strengths and limitations

The coverage afforded by the MBS database allowed analysis of national GP telehealth use by patients and providers for the first time. Linkage of MBS and census data permitted analysis of uptake in relation to a wide range of sociodemographic characteristics and chronic conditions, which has not been reported before. Of the population, 11% did not have a linked census record, which may bias estimates of use; however, as there was complete ascertainment of outcomes among those who linked there is likely to be minimal bias in the relative estimates. Active GPs were identified based on billing information, which may have led to some misclassification. However, 99% of all GP MBS consultations in 2022 were provided by those that we identified as an active GP at least once in 2022. Finally, while interrupted time-series analysis allowed us to quantify trend changes before and after the June 2022 policy change, we advise caution when inferring causality, particularly given the lack of a formal comparison group and the relatively short time series.

### Comparison with existing literature

Similar low use of video by primary care doctors has been observed in other countries (for example, the UK),^13^ although not universally (for example, US).^10^ This is the first study, to our knowledge, assessing the relation of reimbursement or other policies to limit uptake of telephone consultations (absent effects of COVID-19 lockdowns, see Table S7). Regarding equity, policy experts in the US have argued that failing to curtail audio-only consultations risks creating a two-tiered system in which socioeconomically advantaged patients get more options, including in-person and video consultations, while low-income patients are more likely to default to telephone calls.^14,15^ Our data suggest this is not the case in Australia. Rather, most notable is the low use of video but not telephone, regardless of sociodemographic characteristics. However, our finding that older people and socioeconomically disadvantaged populations were less likely to use video is consistent with international experience,^8–10,15^ likely reflecting the ‘digital health divide’, which includes multiple barriers, such as trust and access to technology,^10,16^ as well as provider bias contributing to inequalities in uptake of video.^15,17^

### Implications for research and practice

There are a number of possible explanations for low uptake of video in general practice in Australia and primary care settings in other countries, including issues with access to technology, organisational telemedicine infrastructure,^13,17–20^ and both patient^17,21^ and provider confidence^17,22^ in using virtual care technology. Generally, video has been perceived as logistically challenging, more cognitively demanding and more likely to fail for technical reasons than telephone or in-person modalities.^17^ Furthermore, clinicians feel telephone is adequate for the vast majority of telehealth consultations. For the remainder, the need for hands-on examination explained why video was unsuitable as a replacement for in-person assessment,^13,17^ including for averting serious safety incidents.^23^

That telehealth users living in very remote areas were around 70% more likely to use video than those living in major cities may reflect poorer physical access to GPs in very remote areas, and hence the greater need of a substitute for face-to-face consultations. However, this is not reflected in overall use of telehealth. Alternatively, higher use of video among remote users may reflect historical initiatives to support video use, for example, via models of telehealth consultations with specialists,^24^ which were established in 2011 and augmented in 2019 before the pandemic. Primary care clinics in remote First Nations’ communities have also developed innovative models where patients access remote GPs, using clinic video telehealth infrastructure with the support of local community health workers.^25^ Further, it may reflect more acute presentations in remote areas generally making telephone inappropriate. Higher use of video among patients with dementia may reflect availability of video conferencing in residential aged care facilities.^26^

The research questions and analyses in this study have been directly informed by ongoing engagement with government policymakers and other stakeholders, including GPs and consumers, and the work remains highly relevant to current and future policy considerations. For example, in recognition of the potential benefits of longer telephone consultations at least where there is an established continuing relationship with the patient, the Australian Government has recently reintroduced reimbursement for longer telephone telehealth consultations for patients enrolled with the voluntary patient registration programme introduced in November 2023.^27^ Longer time series would allow assessment of differential impacts of these and other policy and practice changes on overall use of GP services and costs, including differential impacts across subpopulations.

## Data

All data for this project were accessed through the Person Level Integrated Data Asset (PLIDA). The Australian Bureau of Statistics is trusted as the accredited Integrating Authority for PLIDA and provide access to authorised researchers.^11^ The dataset relied on in this article can be accessed via the Australian Bureau of Statistics, subject to the relevant approvals. We have made available all summary data and analysis results in the main results section of supplementary files.
